# Ethnic disparities and temporal trends in health resource allocation: a retrospective decadal analysis in Sichuan, a multi-ethnic Province of Southwest China (2009–2019)

**DOI:** 10.1186/s12913-024-11036-6

**Published:** 2024-04-27

**Authors:** Fang Luo, Yuezhou Huang, Linshan Jiang, Qingqing Fan, Zongchao Gou

**Affiliations:** 1 Department of Pharmacy, West China Hospital, Sichuan University, Chengdu, China; 2 Department of General Surgery, West China Hospital, Sichuan University, Chengdu, China; 3 Breast Disease Center, West China Hospital, Sichuan University, Chengdu, China

**Keywords:** Yi ethnic, Tibetan ethnic, Poverty-stricken region, Theil index, Health practitioner

## Abstract

**Background:**

Research on health resource allocation trends in ethnic minority and impoverished areas in China is limited since the 2009 Medical Reform. This study aimed to investigate the variations and inequalities in health resource distribution among ethnic minority, poverty-stricken, and non-minority regions in Sichuan Province, a multi-ethnic province in Southwest China, from 2009 to 2019.

**Methods:**

The numbers of beds, doctors and nurses were retrospectively sourced from the Sichuan Health Statistics Yearbook between 2009 and 2019. All the 181 counties in Sichuan Province were categorized into five groups: Yi, Zang, other ethnic minority, poverty-stricken, and non-minority county. The Theil index, adjusted for population size, was used to evaluate health resource allocation inequalities.

**Results:**

From 2009 to 2019, the number of beds (Bed_p1000_), doctors (Doc_p1000_), and nurses (Nur_p1000_) per 1000 individuals in ethnic minority and poverty-stricken counties consistently remained lower than non-minority counties. The growth rates of Bed_p1000_ in Yi (140%) and other ethnic minority counties (127%) were higher than in non-minority counties (121%), while the growth rates of Doc_p1000_ in Yi (20%) and Zang (11%) counties were lower than non-minority counties (61%). Doc_p1000_ in 33% and 50% of Yi and Zang ethnic counties decreased, respectively. Nur_p1000_ in Yi (240%) and other ethnic minority (316%) counties increased faster than non-minority counties (198%). The Theil index for beds and nurses declined, while the index for doctors increased. Key factors driving increases in bed allocation include preferential policies and economic development levels, while health practitioner income, economic development levels and geographical environment significantly influence doctor and nurse allocation.

**Conclusions:**

Preferential policies have been successful in increasing the number of beds in health facilities, but not healthcare workers, in ethnic minority regions. The ethnic disparities in doctor allocation increased in Sichuan Province. To increase the number of doctors and nurses in ethnic minority and poverty-stricken regions, particularly in Yi counties, more preferential policies and resources should be introduced.

**Supplementary Information:**

The online version contains supplementary material available at 10.1186/s12913-024-11036-6.

## Background

China is home to 56 ethnic groups, the predominant one being Han, with the remaining 55 constituting various ethnic minority groups [1]. Globally, health resources for ethnic minority populations are often limited [2–4], a trend that is also observable in western China [5–7]. This limited accessibility to health resources often results in poorer health outcomes for minority populations as compared to their non-minority counterparts [8–12]. To address these disparities, the Chinese Government has implemented several measures, including preferential policies for ethnic minority regions. These measures encompass tax reductions and exemptions, as well as support for health infrastructure development [13–15]. A key aim of the medical reform initiated in 2009 was to enhance health resources in ethnic minority regions [16]. However, it remains unclear whether these preferential policies have successfully improved the equitable allocation of health resources between ethnic minority and non-minority regions.

The foundation for delivering high-quality healthcare services is the availability of beds, doctors, and nursing staff within healthcare institutions [17–19]. Factors such as governmental policies, regional economic development, and geographical conditions significantly influence the distribution of these health resources [11, 20, 21]. Ethnic minority populations predominantly reside in remote, high-altitude areas characterized by limited economic development, while the Han population typically inhabits regions with lower altitudes and more robust economies. Consequently, the interplay of policy, geography, and economic factors contributes to disparities in health resource allocation. The impact of preferential policies and the influence of adverse geographical and economic factors on the provision of beds, doctors, and nurses in ethnic minority regions following the 2009 medical reform remain to be fully understood.

Sichuan Province, located in southwest China, is home to all 56 ethnic groups found within the country. The majority of the 55 ethnic minority populations reside in 67 of the 181 counties in the province. These 67 ethnic autonomous counties account for 62.1% of Sichuan's total area, with an average altitude exceeding 2400 m above sea level (a.s.l.) and a per capita gross domestic product (GDP) lower than that of non-minority regions. The unique combination of natural and social characteristics makes Sichuan an ideal location for investigating the effects of policies, geographical environment, and economic development on the temporal variations and disparities of health resources among distinct ethnic groups.

In response to the scarcity of healthcare resources in ethnic minority counties, the Sichuan Province Government implemented the "Ten-year Action Plan for Health Development in Ethnic Minority Counties of Sichuan Province" from 2011 to 2020 [22]. The execution of this action plan resulted in an investment exceeding 2 billion RMB to increase the number of healthcare facilities and practitioners in these minority regions. We hypothesize that the growth rate of healthcare resources (beds, doctors, and nurses) in ethnic minority counties has surpassed that of non-minority counties since 2009. However, the per capita resources in ethnic minority counties likely remain inferior to those in non-minority counties due to the significantly low baseline values in the former. Furthermore, we propose that the equitable distribution of healthcare resources improved between 2009 and 2019. We also speculate that policy measures and economic development primarily drive variations in healthcare facilities, while income, economic development, and geographical factors significantly influence the allocation of healthcare practitioners. Specifically, we aim to investigate the changes and disparities in health resource allocation among ethnic minority, poverty-stricken, and non-minority regions in Sichuan Province from 2009 to 2019, and to identify the key factors influencing these changes.

## Methods

### County demographics

**Fig. 1:**
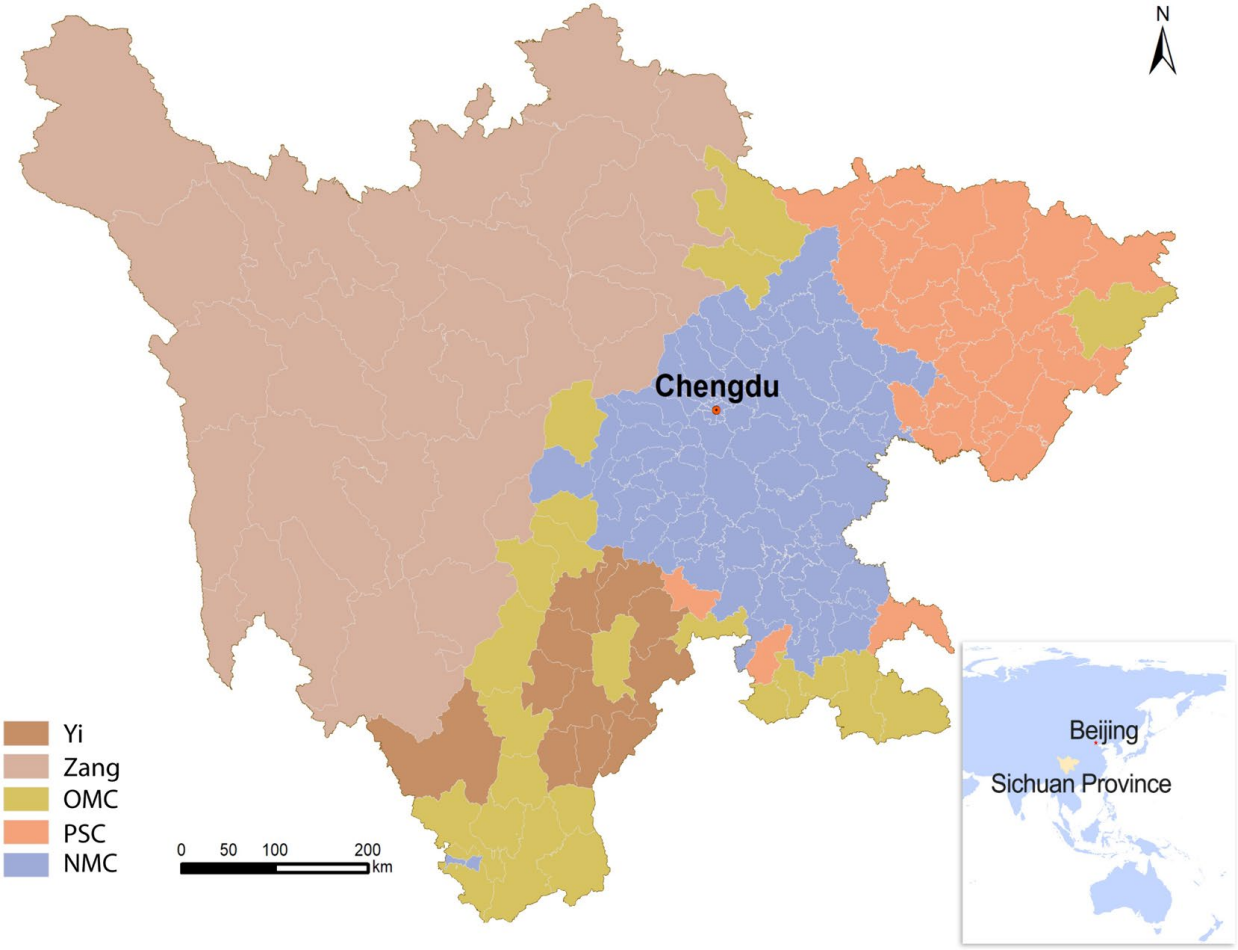
The location of 181 counties in Sichuan Province. Yi, Zang, OMC, PSC and NMC refer to Yi, Zang and Other ethnic minority, Poverty-stricken and Non-minority counties, respectively. Chengdu City is the capital of Sichuan Province. Abbreviations: NMC, non-minority county; OMC, other minority ethnic county; PSC, poverty-stricken county

Sichuan, a province in Southwest China, is characterized by its diverse multi-ethnic composition, which includes 181 counties. Approximately 5.7 million individuals belong to ethnic minority groups, representing 6.80% of the province's total population. These individuals predominantly reside in the western region of Sichuan. Among the 55 ethnic minority groups, the Yi and Zang (Tibetan) constitute the largest (55.2%) and second largest (29.6%) populations, respectively. According to an official document from the Sichuan Province Government, the 67 ethnic minority counties were classified into three categories: Yi (12 counties), Zang (32 counties), and other ethnic minority counties (23 counties). The remaining 114 non-minority counties were divided into two categories: poverty-stricken counties (PSC, 32 counties) and non-minority counties (NMC, 82 counties) as shown in Fig. 1. PSCs, characterized by low economic development, have implemented policies similar to those in ethnic minority counties since 2010. The classification of all categories, including Yi, Zang, and other ethnic minority counties, was established by the National Ethnic Affairs Commission of China. Similarly, the PSC and NMC designations were classified by the Leading Group Office of Poverty Alleviation and Development of China. Notably, the preferential policies and investments mentioned above were exclusively directed towards minority counties and PSC counties. Given our objective to examine the impact of these preferential policies on changes in health resources, we adhere to the government's classification system for our analysis.

### Data and calculation

Data relating to the number of beds, health practitioners, doctors, nursing staff, and average income of healthcare practitioners across counties in Sichuan Province were obtained from the Sichuan Health Statistics Yearbook between 2009 and 2019 [23]. The term "beds" encompasses those within various health institutions. The term "doctors" encompasses licensed physicians and physician assistants; does not include village doctors. Furthermore, the category of nursing staff comprises solely registered nurses. Due to the difficulty in accessing income data for doctors and nurses at the county level during this period, the average income of healthcare practitioners in each county was employed as an economic indicator. Sixteen to 31% of healthcare practitioners are pharmacists and clinical lab technicians [23]. The Gross Domestic Product (GDP) per capita for each county, sourced from the Sichuan Statistics Yearbook, served as an additional economic indicator. To account for geographical environment, the average altitude of each county was utilized as a proxy, with data obtained from topographic-map.com.

To characterize the distribution of health resources between 2009 and 2019, the number of beds (Bed_p1000_), doctors (Doc_p1000_), and nurses (Nur_p1000_) per 1000 persons in each county was assessed. The growth rate of Bed_p1000_, Doc_p1000_, and Nur_p1000_ was employed to describe variations in health resources, as determined by the following equation.

1 $$\mathrm{Growth}\;\mathrm{rate}=\left({\text{H}}_{2019}-{\text{H}}_{2009}\right)/{\text{H}}_{2009}$$

where H_2019_ refers to Bed_p1000_, Doc_p1000_ or Nur_p1000_ of each county in 2019. H_2009_ refers to Bed_p1000_, Doc_p1000_ or Nur_p1000_ of each county in 2009.

We also used DNpB and DN/HP as two proxies to describe the difference in variation in Bed_p1000_, Doc_p1000_ and Nur_p1000_.

2 $$\mathrm{DNpB }= ({{\text{Doc}}}_{{\text{p}}1000} + {{\text{Nur}}}_{{\text{p}}1000}) / {{\text{Bed}}}_{{\text{p}}1000}$$

3 $${\text{DN}}/\mathrm{HP }= ({{\text{Doc}}}_{{\text{p}}1000} + {{\text{Nur}}}_{{\text{p}}1000}) / {{\text{HP}}}_{{\text{p}}1000}$$

where HP _p1000_ refers to the number of all health practitioners per 1000 persons in each county.

The Theil index (TI) was utilized as a metric to assess the disparities in health resource distribution across various categories by previous researches [5–7, 20, 24, 25]. To enable a comprehensive analysis of overall equity, the TI was segregated into two components: intra-category and inter-category. Correspondingly, the TI values for both intra-category and inter-category were calculated employing the subsequent formula.

4 $${\text{TI}}= \sum\nolimits_{i=1}^{n}{p}_{i}Ln(\frac{{p}_{i}}{{y}_{i}})$$

where ${p}_{i}$ is the proportion of the population in a county relative to the total population in Sichuan Province, ${y}_{i}$ is the proportion of health resources (beds, doctors and nurses) in a county relative to the total number in Sichuan Province. *n* refers to the number of counties in Sichuan Province. *i* refers to the ith county in the province.

TI can be divided into TI_*intra*_ and TI_*inter*_, and they can be calculated as follows:

5 $$\mathrm{TI }= {{\text{TI}}}_{intra}+ {{\text{TI}}}_{inter}$$

6 $${{\text{TI}}}_{intra}= \sum\nolimits_{j=1}^{k}{p}_{j}{TI}_{j}$$

7 $${{\text{TI}}}_{intra}= \sum\nolimits_{j=1}^{k}{p}_{j}Ln(\frac{{p}_{j}}{{y}_{j}})$$

where ${p}_{j}$ is the proportion of the population in a category relative to the total population in Sichuan Province, ${y}_{g}$ is the proportion of health resources (beds, doctors and nurses) in a category relative to the total number in Sichuan Province. *k* refers to the number of categories (5 categories). *j* refers to the jth category in the province.

### Statistics

Utilizing a linear regression model, this study aimed to evaluate the influence of income, GDP per capita, and altitude on the Bed_p1000_, Doc_p1000_ and Nur_p1000_ in each county. To ensure the validity of the linear regression models, three assumptions were examined: normality, equal variances on the residuals, and independence. A one-way analysis of variance (ANOVA) was employed to test the deviation between the slope of each linear regression model and zero, with a significance level set at 0.05.

## Results

### Number and growth rates of the Bed_p1000_, Doc_p1000_ and Nur_p1000_ from 2009 to 2019

**Fig. 2:**
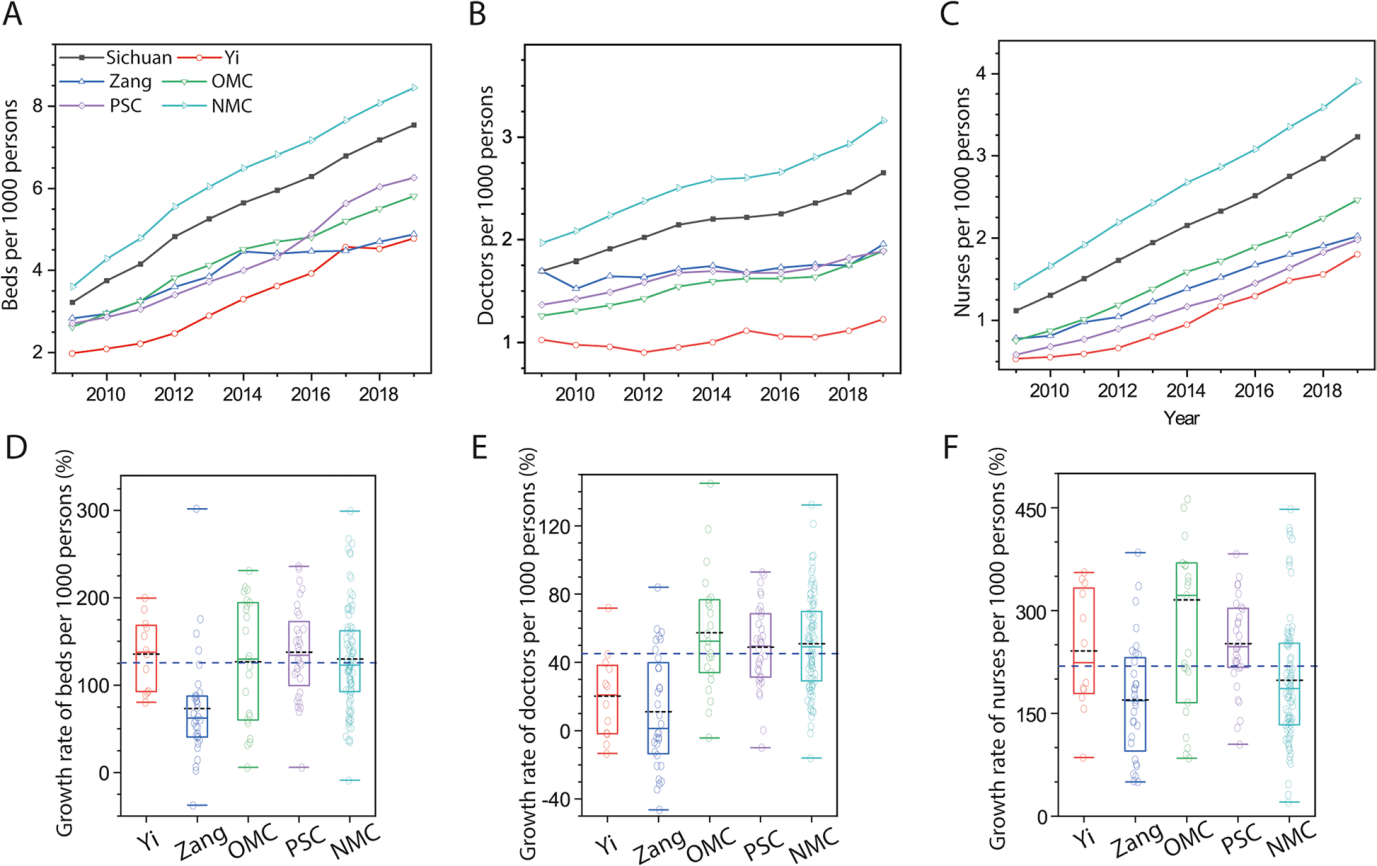
Trends in the number of beds (**A**), doctors (**B**) and nurses (**C**) per 1000 persons in Sichuan, a typical multi-ethnic province in southwest China from 2009 to 2019. Growth rates of the number of beds (**D**), doctors (**E**) and nurses (**F**) per 1000 persons in Sichuan. The blue dashed line represents the average growth rate of Sichuan Province. Yi, Zang, OMC, PSC and NMC refer to Yi, Zang and Other ethnic minority, Poverty-stricken and Non-minority counties, respectively. Abbreviations: NMC, non-minority county; OMC, other minority ethnic county; PSC, poverty-stricken county

From 2009 to 2019, the Bed_p1000_, Doc_p1000_ and Nur_p1000_ values in Yi, Zang, and other ethnic minority and poverty-stricken counties consistently remained lower than those in non-minority counties and the Sichuan Province average (Fig. 2 A-C). Among these, the Yi ethnic minority counties exhibited the lowest values across all three indicators. The Doc_p1000_ ratio in Yi counties compared to the Sichuan Province average decreased from 61% in 2009 to 46% in 2019, while the Bed_p1000_ increased from 59 to 63% and the Nur_p1000_ increased from 48 to 56%. Notably, non-minority counties were the only category to consistently display higher Bed_p1000_, Doc_p1000_ and Nur_p1000_ values than the Sichuan Province average throughout the study period.

Although all five categories experienced an increasing trend since 2009, the mean growth rates of Bed_p1000_, Doc_p1000_ and Nur_p1000_ varied among them (Fig. 2 D-F). The Zang counties exhibited the lowest growth rates across all three indicators among all categories. Approximately 88%, 91%, and 63% of Zang counties demonstrated lower growth rates in Bed_p1000_, Doc_p1000_ and Nur_p1000_, respectively, compared to the Sichuan Province average from 2009 to 2019. Alarmingly, around 50% of Zang counties experienced a negative growth rate in Doc_p1000_. The Yi counties displayed the second-lowest mean Doc_p1000_ growth rate, with approximately 33% of these counties exhibiting a decrease in 2019 compared to 2009. In contrast, the mean growth rates of all three indicators in the Other ethnic minority and Poverty-stricken counties exceeded the Sichuan Province average (Fig. 2 D-F).

### Number of doctors and nurses per bed

**Fig. 3:**
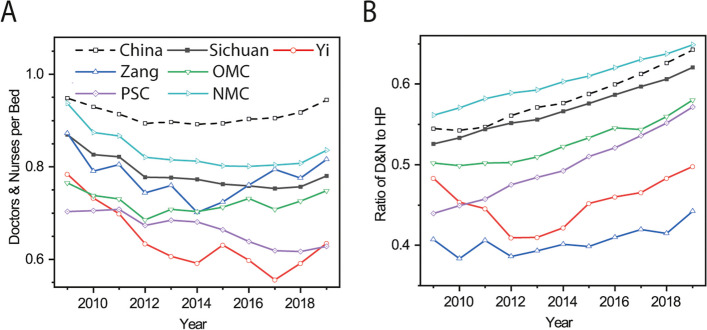
Trends in the ratio of the sum of doctors and nurses per 1000 persons to the beds per 1000 persons (**A**) and the sum of doctors and nurses per 1000 persons to the number of health practitioners per 1000 persons (**B**) in Sichuan from 2009 to 2019. Yi, Zang, OMC, PSC and NMC refer to Yi, Zang and Other ethnic minority, Poverty-stricken and Non-minority counties, respectively. Abbreviations: NMC, non-minority county; OMC, other minority ethnic county; PSC, poverty-stricken county

In 2019, the number of doctors and nurses per bed (DNpB) of the three ethnic minority and poverty-stricken counties was lower than in 2009, despite varying temporal processes of change across the 11-year period (Fig. 3A). The Yi counties experienced the largest decline in DNpB (19%) compared to the other four categories. By 2019, the DNpB of the Yi counties constituted approximately 81% of the Sichuan Province average. Interestingly, the Zang counties displayed a higher DNpB than the Sichuan Province average from 2017 to 2019. The DN/HP of the three ethnic minority and poverty-stricken counties consistently remained lower than that of the non-minority counties (Fig. 3B). The Zang and Yi counties exhibited the lowest and second-lowest DN/HP values among the five categories of counties, respectively.

### Theil index

**Fig. 4:**
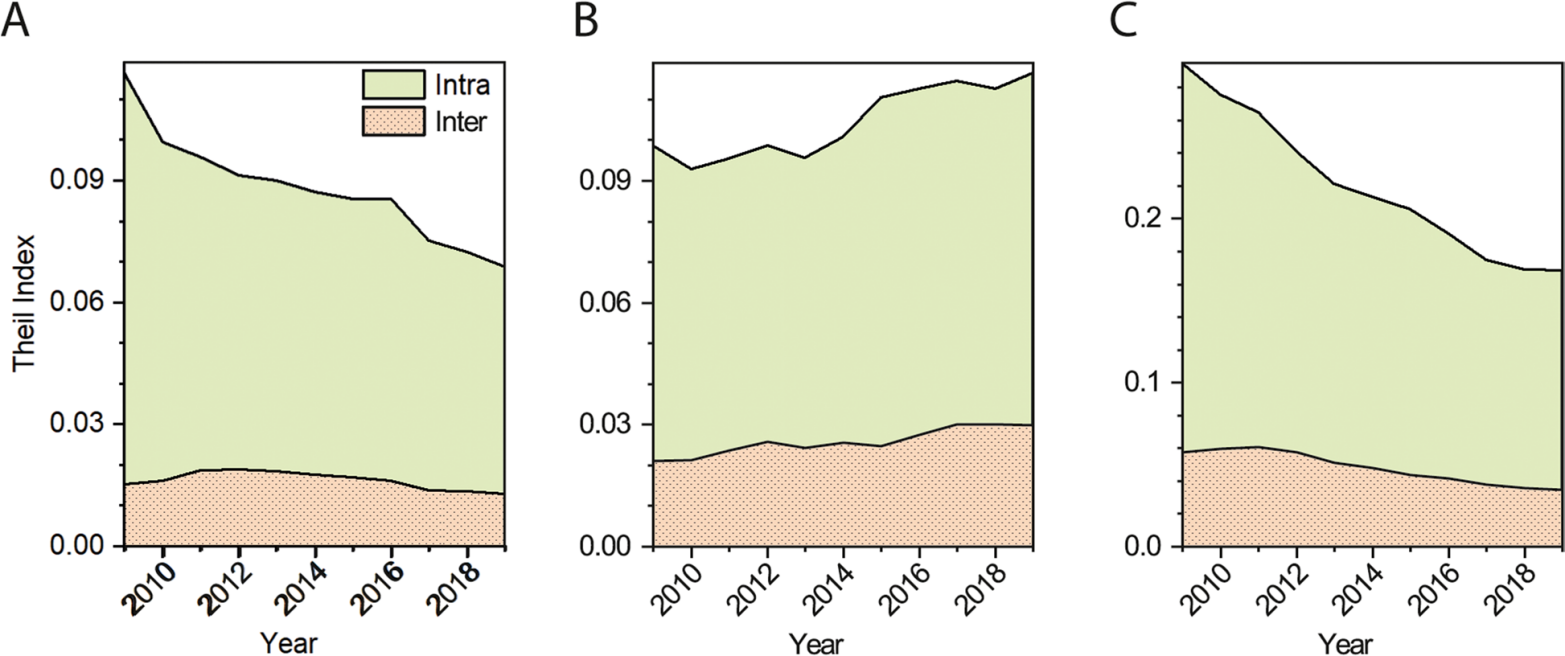
Trends in the Theil index of beds (**A**), doctors (**B**) and nurses (**C**) in Sichuan from 2009 to 2019. The Intra and Inter refer to the contribution of intra-counties and inter-counties to the Theil index, respectively

The Theil Index (TI) of beds and nurses demonstrated a decreasing trend from 2009 to 2019, while the TI of doctors exhibited an increasing trend (Fig. 4). The TI of nurses consistently remained higher than that of beds and doctors. The contribution of inter-category factors to the doctor's TI increased from 2009 to 2019, although intra-category contributions constituted over 70% of the TI.

### Correlations between health resources and economic indicators

**Fig. 5:**
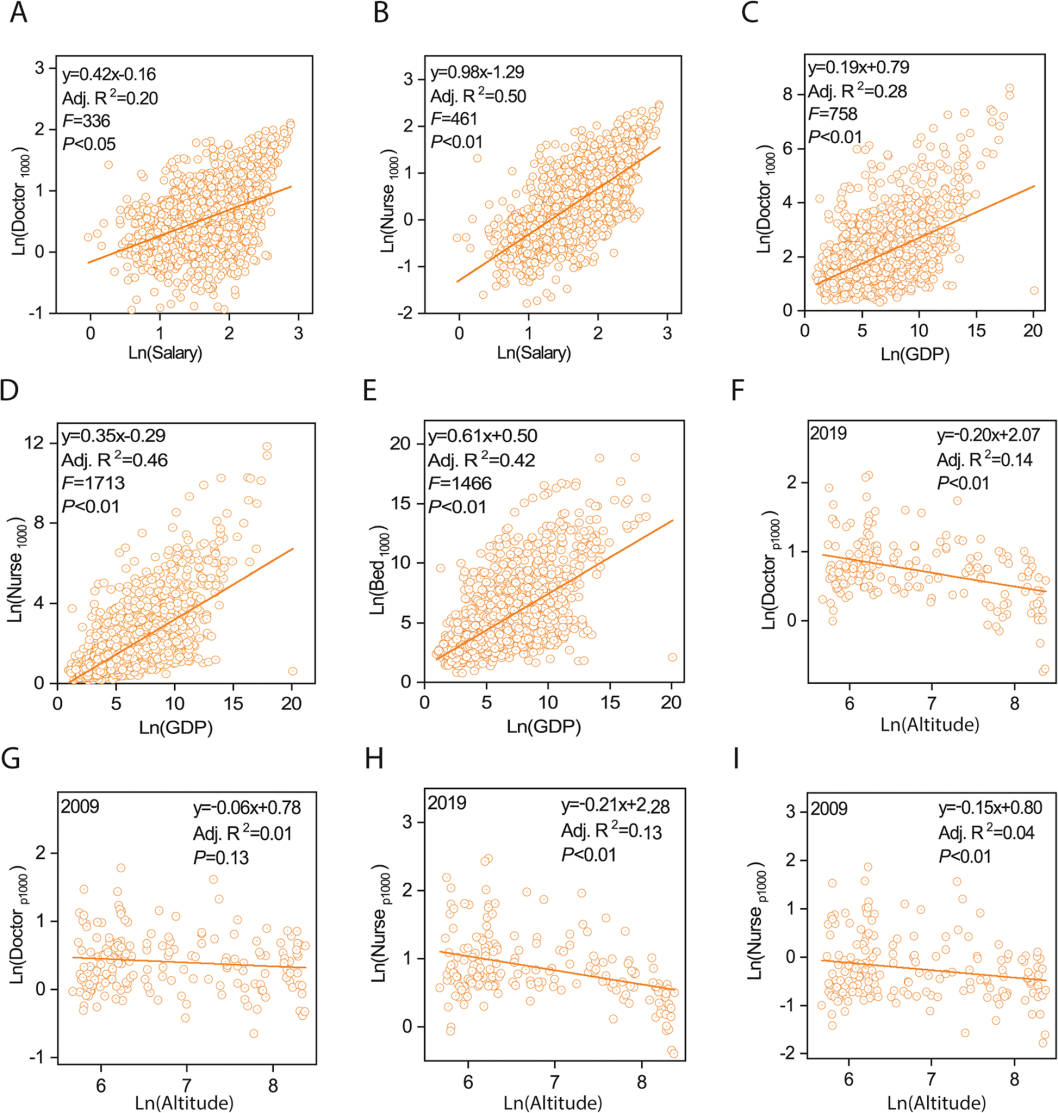
The linear regression models between the average annual income of health practitioners and the number of doctors per 1000 persons (**A**) and nurses per 1000 persons (**B**) in Sichuan counties from 2009 to 2019 (*n* = 1991). The linear regression models between the gross domestic product per capita (GDP) and the number of doctors per 1000 persons (**C**), nurses per 1000 persons (**D**) and beds per 1000 persons (**E**) of each Sichuan county from 2009 to 2019 (*n* = 1991). The linear regression models between average altitude and the number of doctors per 1000 persons (F, 2019; G, 2009) and the nurses per 1000 persons (H, 2019; I, 2009) of each county in Sichuan (*n* = 181)

Both the Doc_p1000_ and Nur_p1000_ values exhibited positive correlations with the average annual income of health practitioners from 2009 to 2019 (Fig. 5A, B). However, the model only predicted 20% of the variance in Doc_p1000_, while approximately 50% of the variance in Nur_p1000_ was predicted. Similarly, a greater proportion of the variance in Nur_p1000_ was explained by each county's GDP per capita compared to Doc_p1000_ (Fig. 5C, D). The Bed_p1000_ also displayed a positive correlation with GDP per capita, with the linear regression model predicting approximately 42% of the variance in Bed_p1000_ (Fig. 5E). In 2019, the Doc_p1000_ and Nur_p1000_ values were significantly negatively correlated with each county's average altitude, while their correlations in 2009 were not significant (Fig. 5 F-I).

## Discussion

Our findings confirm that health resources (Bed_p1000_, Doc_p1000_ and Nur_p1000_) are less distributed in ethnic minority counties. Despite lower Bed_p1000_ values in all ethnic minority counties compared to Non-minority counties, our results demonstrate that preferential policies have effectively enhanced health facilities (beds), rather than healthcare workers, in ethnic minority regions. This is evidenced by the higher ratio of Bed_p1000_ than Doc_p1000_ and Nur_p1000_ between ethnic minority and Non-minority counties in 2019. Additionally, the reduced Theil Index (TI) for beds from 2009 to 2019 (Fig. 4A) also shows the roles of policies in increasing beds in the ethnic minority counties. This is further supported by the decrease in doctors in many Yi and Zang counties (Fig. 2E). Indeed, it is easier to build hospitals in underserved areas than it is to staff them.

Our findings do not support a higher growth rate of health resources in all ethnic minority counties compared to Non-minority counties (Fig. 2), highlighting the inadequacy of current policies in augmenting the doctor population in these ethnic minority regions. This is firstly evidenced by the negative growth of doctors in approximately 33% of Yi and 50% of Zang counties. The deficiency is further supported by the low DNpB in the Yi group and DN/HP values for both Yi and Zang counties (Fig. 3). The higher DNpB in Zang counties relative to the Sichuan Province average between 2017 and 2019 does not imply adequate health practitioner and facilities. Rather, it demonstrates insufficiency in both areas as the number of doctors, nurses and beds in the Zang counties only accounted for 70%, 61% and 79% of the Sichuan Province average during the three years, respectively. In addition, the rising inter-category TI for doctors (Fig. 4) suggests the widening disparities in doctor distribution between different ethnic regions. Wang & Pan similarly reported difficulties in accessing doctors for ethnic minority groups due to shortages in these regions [26]. As health practitioners play a critical role in determining health system performance and outcomes [24, 27], this shortage may have adverse consequences. For example, maternal mortality ratios in Zang and Yi ethnic minority counties exceeded those in non-minority counties, as reported in the Sichuan Health Statistical Yearbook from 2009 to 2019.

Our analysis partially corroborates a decline in the equity of health resource allocation in Sichuan Province between 2009 and 2019. This is evident as the Theil Index (TI) for beds and nurses decreased, while the TI for doctors displayed a contrasting trajectory (Fig. 4). The observed decrease in TI for beds and nurses represents different forms of equity. For instance, the Bed_p1000_ across all five categories exceeded that in Guangdong Province in 2019 (4.73), which has the highest GDP in China. This underscores a high level of equality in bed allocation. Conversely, all ethnic minority and PSC counties demonstrated a significantly lower Nur_p1000_ compared to the average in western China in 2019 (3.26), generally considered the least developed region in China. Hence, the distribution of nurses among the five categories exemplifies a low level of equality. The declining equity in doctor allocation suggests that the existing preferential policies have not effectively bolstered the numbers of doctors in ethnic minority counties. It's plausible that other factors contribute to this diminished equity in doctor allocation.

Economic development considerably influenced the increase in beds between 2009 and 2019. Approximately 42% of the variation in Bed_p1000_ was accounted for by GDP per capita (Fig. 5). GDP per capita, a key economic development indicator, is widely recognized as a significant influence on health resources, as evidenced by numerous previous studies [25, 28–31]. Although non-minority counties generally have higher GDP per capita than ethnic minority counties, the more substantial growth rate of Bed_p1000_ in Yi and other ethnic minority counties compared to non-minority counties attests to the effectiveness of preferential policies in increasing bed availability in these ethnic minority counties (Fig. 2). Thus, we conclude that preferential policies and economic development over the past decade have been crucial in enhancing bed availability in ethnic minority regions, thereby promoting health facility equity in Sichuan Province.

The significantly lower health practitioner growth rates (Nur_p1000_ and Doc_p1000_) in Zang and Yi counties compared to other ethnic minority counties cannot be solely ascribed to economic indicators (Fig. 5). Other ethnic minority counties exhibited only slightly higher GDP per capita than Zang counties, as per the Sichuan Statistical Yearbook. Considering the higher average altitudes of Yi (2139 m a.s.l.) and Zang (3656 m a.s.l.) counties compared to other ethnic minority counties (1592 m a.s.l.), challenging geographical environments could be an additional factor hindering the growth of doctors and nurses in Yi and Zang counties. This is further supported by the finding that correlations between health practitioner metrics (Nur_p1000_ and Doc_p1000_) and average county altitude became significant in 2019, having been insignificant in 2009 (Fig. 5). Consequently, the relatively low economic development levels and harsh geographical environments in Yi and Zang counties contribute to the lower growth rate of doctors in these regions compared to the other three regions, ultimately leading to decreased equity in doctor availability across Sichuan Province (Fig. 4). Given the complexities associated with swiftly altering geographical circumstances and improving the economic status of the Zang and Yi counties, we propose two potential strategies to address the shortage of healthcare practitioners in these areas. Firstly, we recommend offering additional financial incentives for physicians and nursing staff working in these regions. Secondly, we suggest promoting internet-enabled diagnostic and therapeutic services to mitigate the current shortage of medical professionals in the remote Zang and Yi counties.

Our study has several limitations. Language barriers and a relatively low level of primary education may influence residents' health-seeking behavior and medical graduates' willingness to serve in ethnic minority regions. Owing to data accessibility challenges, we did not analyze the impact of these two factors on the trend in Nur_p1000_ and Doc_p1000_. Additionally, a questionnaire survey is crucial to elucidate the reasons for the shortage of health practitioners in ethnic minority regions, particularly in the Yi and Zang regions. Furthermore, as the primary goal of this study was to highlight disparities between ethnic minority and non-minority counties, counties defined by government documents as both ethnic minority and poverty-stricken were classified as ethnic minority counties in this study. However, this mutually exclusive categorization is unlikely to significantly affect the results, as both types of counties received similar preferential policies from 2010 to 2019.

## Conclusions

To address disparities in health resources between ethnic minority and non-minority regions, both the central and local governments in China have implemented a range of preferential policies over several decades. Our findings suggest that these measures have successfully increased the number of beds in healthcare facilities. However, these policies have not been as effective in boosting health practitioner resources in ethnic minority counties. Furthermore, our results indicate that the challenging geographical environment and relatively low levels of economic development in the Zang and Yi counties are two primary factors hindering the growth of medical professionals. We propose the enhancement of internet-enabled diagnostic and therapeutic services to address the shortage of licensed doctors in the remote Zang and Yi counties in the short term. In the long term, more preferential policies should be introduced to attract more health practitioners to work in ethnic minority counties.

### Supplementary Information

**Supplementary Material 1.**

## Data Availability

All data analyzed during this study are included in the supplementary.
